# Intravenous iron therapy among patients with heart failure and iron deficiency: An updated meta-analysis of randomized controlled trials

**DOI:** 10.1016/j.heliyon.2023.e17245

**Published:** 2023-06-15

**Authors:** Mohamed Hamed, Sheref A. Elseidy, Asmaa Ahmed, Ravi Thakker, Hend Mansoor, Houman Khalili, Amr Mohsen, Mamas A. Mamas, Subhash Banerjee, Dharam J. Kumbhani, Islam Y. Elgendy, Ayman Elbadawi

**Affiliations:** a Department of Internal Medicine, Florida Atlantic University, Boca Raton, FL, USA; b Department of Internal Medicine, Rochester General Hospital, Rochester, NY, USA; c Division of Cardiology, University of Texas Medical Branch, Galveston, TX, USA; d Department of Pharmacy, University of Kentucky, Lexington, KY, USA; e Division of Cardiology, Memorial Healthcare System, Hollywood, FL, USA; f Division of Cardiology, Loma Linda University, Loma Linda, CA, USA; g Keele Cardiovascular Research Group, Keele University, Keele, UK; h Division of Cardiology, Baylor University Medical Center, Dallas, TX, USA; k Division of Cardiology, University of Texas Southwestern Medical Center, Dallas, TX, USA; j Division of Cardiovascular Medicine, Gill Heart Institute, University of Kentucky, Lexington, KY, USA

**Keywords:** IV iron, Iron deficiency, Anemia

## Abstract

**Background:**

Randomized clinical trials (RCTs) evaluating the role of intravenous (IV) iron administration in patients with heart failure (HF) and iron deficiency (ID) have yielded inconsistent results.

**Methods:**

Electronic search of MEDLINE, EMBASE and OVID databases was performed until November 2022 for RCTs that evaluated the role of IV iron administration in patients with HF and ID. The main study outcomes were the composite of HF hospitalization or cardiovascular mortality, and individual outcome of HF hospitalization. Summary estimates were evaluated using random effects model.

**Results:**

The final analysis included 12 RCTs with 3,492 patients (1,831 patients in the IV iron group and 1,661 patients in the control group). The mean follow-up was 8.3 months. IV iron was associated with a lower incidence in the composite of HF hospitalization or cardiovascular mortality (31.9% vs. 45.3%; relative risk [RR] 0.72; 95% confidence interval [CI] 0.59–0.88) and individual outcome of HF hospitalization (28.4% vs. 42.2; RR 0.69; 95% CI 0.57–0.85). There was no significant difference between both groups in cardiovascular mortality (RR 0.88; 95% CI 0.75–1.04) and all-cause mortality (RR 0.95; 95% CI 0.83–1.09). IV iron was associated with lower New York Heart Association class and higher left ventricular ejection fraction (LVEF). Meta-regression analyses showed no effect modification for the main outcomes based on age, hemoglobin level, ferritin level or LVEF.

**Conclusion:**

Among patients with HF and ID, IV iron administration was associated with reduction in the composite of HF hospitalization or cardiovascular mortality and driven by a reduction in HF hospitalization.

## 1 Introduction

Iron deficiency (ID) is a common co-morbidity among patients with heart failure (HF) [1,2]. It is reported that ID is prevalent up to 30–50% of stable chronic HF patients and up to 50–80% among those with acute HF [1]. The association between HF and ID is hypothesized to be related to the decrease in iron absorption, depletion of iron stores and reduced availability of the recycled iron from the reticuloendothelial system among patients with HF [3,4]. With the known important role of iron in oxygen storage, oxygen transport and aerobic metabolism in skeletal muscles, ID may contribute to fatigue, dyspnea, and the exercise intolerance in HF patients [1,3,5]. Irrespective of anemia, ID is suggested to increase mortality and worsen prognosis in HF patients; hence iron therapy has been studied as a potential treatment for HF [5]. Intravenous (IV) iron may be the preferred treatment for iron deficiency in only certain medical conditions due to its possible allergic/anaphylactic side effects [[6], [7], [8]]. Although multiple studies have studied the effect of IV iron on improving the clinical outcomes of HF and reducing the risk of HF hospitalization, these studies have yielded inconsistent results [3,7,[9], [10], [11], [12], [13], [14], [15], [16], [17], [18], [19], [20], [21], [22], [23], [24], [25], [26], [27], [28], [29], [30]]. In the recent IRONMAN (Effectiveness of Intravenous Iron Treatment versus Standard Care in Patients with Heart Failure and Iron Deficiency) trial, IV iron among patients with HF and ID did not reduce the risk of HF hospitalization or cardiovascular mortality compared with usual care [18]. Thus, we sought to conduct a meta-analysis of the available randomized controlled trials (RCTs) to evaluate the efficacy of IV iron administration on patients with HF and ID.

## 2 Methods

### 2.1 Data sources and search strategy

A comprehensive electronic database search of MEDLINE, Cochrane, and OVID was performed through November 2022, using the search terms “iron deficiency” OR “intravenous iron” OR “iron therapy” AND “heart failure” for RCTs that evaluated the efficacy of IV iron administration in improving clinical outcomes in patients with HF and ID. Further screening of the bibliographies of the retrieved articles, earlier meta-analyses, and ClinicalTrials.gov to identify any other pertinent studies. This systematic review and meta-analysis was conducted in accordance with Preferred Reporting Items for Systematic Reviews and Meta-Analyses (PRISMA) guidelines [31] (Supplemental Table 1). The protocol for this meta-analysis and systematic review was prospectively registered at PROSPERO **(ID 388733).**

### 2.2 Selection criteria

This study included RCTs that evaluated the role of IV iron administration compared with control in patients with HF and ID. Included studies enrolled patients with ID regardless of the presence of anemia. Only English-language studies with full results published until November 2022 were included. Studies which did not report clinical outcomes were excluded.

### 2.3 Data extraction

The following data were independently extracted from the included studies by two investigators (SAE and MH): study design, baseline characteristics and clinical outcomes. Disagreements between investigators were resolved by consensus.

### 2.4 Outcomes

The main study outcomes were the composite of HF hospitalization or cardiovascular mortality and the individual outcome of HF hospitalization. Other outcomes included cardiovascular mortality, all-cause mortality, improvement of left ventricular ejection fraction (LVEF), New York Heart Association (NYHA) class and 6-min walk (6 MW) test. Clinical outcomes were reported using an intention-to-treat basis at the longest reported follow-up period.

### 2.5 Assessment of the quality of the included studies

The quality of the included trials and risk of bias was assessed using the Cochrane bias risk assessment tool, which comprises 7 criteria: random sequence generation, allocation concealment, blinding of participants and personnel, blinding of outcome assessment, incomplete outcome data, selective reporting, and other sources of bias [32]. Then studies were classified into high risk, low risk or unclear risk of bias (Supplemental Table 2).

### 2.6 Statistical analysis

Random effects model utilizing Mantel-Haenszel method was used to pool data and generate estimates of the treatment effect. I^2^ statistic was used to assess the heterogeneity among the included trials. I^2^ statistic values of <25%, 25% to 50%, and >50% were considered to be a low, moderate, and a high degree of heterogeneity, respectively [32]. A subgroup analysis for acute versus chronic HF was performed. The following sensitivity analyses were conducted: including studies that used IV ferric carboxymaltose (FCM) (i.e., the most frequently used IV iron formulation), excluding studies with higher risk of bias, including only studies with >100 sample size at each group, and including only studies with follow-up >3 months. Also, stepwise sensitivity analyses were conducted by excluding one study at a time to evaluate the study with highest contribution to heterogeneity in the main study outcomes. Meta-regression analyses were conducted to evaluate the effect modification of main outcomes based on age, sex, hemoglobin level, ferritin level and LVEF. We reported the outcomes as risk ratios (RR) for categorical variables and mean differences (MD) for continuous variables. P-values <0.05 were considered statistically significant. Funnel plot was used to assess publications bias [33]. RevMan 5.4 software (Cochrane Collaboration, Oxford, UK) was used to conduct all the statistical analyses.

## 3 Results

### 3.1 Included studies

**Fig. 1:**
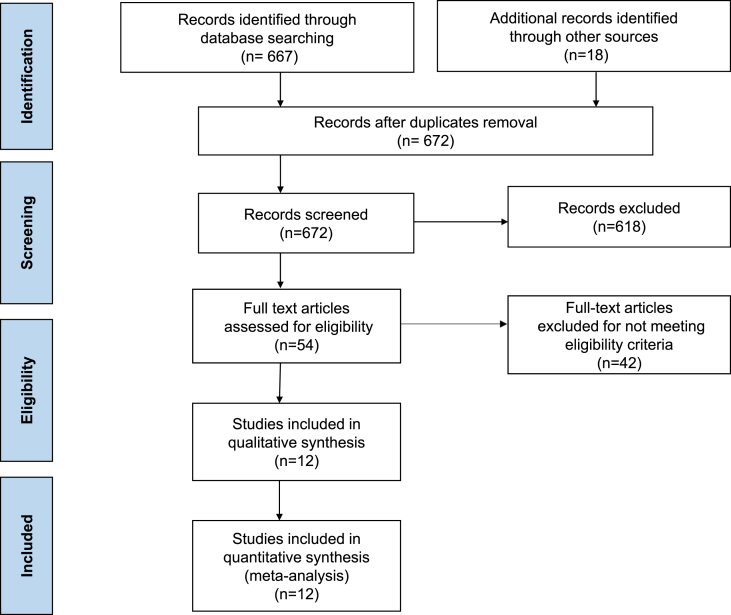
Study flowsheet.

The study selection process is outlined in Fig. 1. Our final analysis included 12 RCTs with a total of 3,492 patients [3,7,[9], [10], [11], [12], [13], [14], [15], [16], [17], [18]]: 1,831 patients in the IV iron group and 1,661 patients in the control group. Baseline characteristics of the included studies and study population were outlined in Tables 1, 2, and 3. The mean follow-up duration was 8.3 months. The mean age was 70.5 years and the proportion of men was 61.3%. The LVEF varied among the included studies, but most studies included patients with LVEF ≤45% [3], [9], [10], [11], [12], [13], [15], [16], [17], [18] except IDAN-HF and PRACTISE-ASIA-HF which included patients with clinical HF regardless of LVEF [7,14]. Most of the studies included patients with chronic HF [3,7,[9], [10], [11], [12], [13],^16^,^17^], while AFFIRM-AHF and PRACTISE-ASIA-HF enrolled patients with acute HF [14,15]. IRONMAN involved patients with both chronic and acute HF [18]. The included studies used various preparations and doses of IV iron. Six studies used FCM. [3,[12], [13], [14], [15], [16]], 3 used iron sucrose [[9], [10], [11]], IDAN-HF used iron dextran [7], FERRIC-HF II used iron isomaltoside [17], and IRONMAN used ferric derisomaltose [18]. Toblli et al., IDAN-HF and FERRIC-HF II were single centered studies [7,9,17], while all other studies were multi-center trials [3,[10], [11], [12], [13], [14], [15], [16],^18^]. The quality of included studies is presented in Supplemental Table 2. Most of the studies included were double-blinded except FERRIC-HF, EFFECT-HF, PRACTISE-ASIA-HF and IRONMAN, which were open-label studies [10,13,14,18]. Otherwise, all studies were deemed to be at low risk of bias.

**Table 1:** Characteristics of the included studies.

Study	Year of publication	no of centers	Group 1 (IV iron)	Group 2 (control)	Longest follow up duration	Inclusion criteria	Primary outcome	Definition of Iron deficiency	Iron formula and dosing
IRONMAN	2022	Multi-centered	569	568	5.4 years	- Age ≥18 years with heart failure (LVEF ≤45%) - Current or recent (within 6 months) admission to hospital due to HF, for patients not fulfilling either of these criteria (NT-proBNP >250 ng/L in sinus rhythm or >1000 ng/L in atrial fibrillation, or BNP >75 ng/L in sinus rhythm or >300 ng/L in atrial fibrillation	All hospital admissions for heart failure and cardiovascular death analyzed using a recurrent events analysis	TSAT <20% or serum ferritin <100 μg/L	- Ferric derisomaltose was given based on hemoglobin value and bodyweight. - Average dose was 20 mg/kg, up to 2000 mg. - Patients received intravenous ferric derisomaltose at trial visits (4 weeks after randomization and every 4 months) if ferritin <100 μg/L or if ferritin ≤400 μg/L and TSAT was <25%.
IRON-CRT	2021	Multi-centered	37	38	3 Months	- Age ≥18 years - Had stable HF - Received CRT for HFrEF >6 months - Had persistently reduced LVEF <45% - ≥ 98% biventricular pacing the last 6 months - NYHA class ≥ II - ID	MACEs including death, hospitalization for ACS, hospitalization for HF, hospitalization for stroke	Serum ferritin <100 ng/mL or serum ferritin 100–300 ng/mL if TSAT <20%.	- FCM ranging between 500 and 2000 mg for one time dosing with no maintenance therapy
IDAN-HF	2021	Single centered	30	30	6 months	- Adults>18 years who followed up for HF in the clinic for at least 6 months	6 MW test	TSAT <20%	- 1,000 g of parenteral iron dextran infusion given in 3–5 divided doses over 3 to 5 weeks.
AFFIRM-AHF	2020	Multi-centered	558	550	12 months	- Age ≥18 years - With prior hospitalization for acute HF - Concomitant ID - LVEF ≤50%.	the total HF hospitalizations and cardiovascular death	Ferritin <100 μg/L, or 100–299 μg/L with TSAT <20%)	- 500 to 1000 mg iron (FCM) at index hospitalization and at week 6. - The subsequent maintenance doses were given at weeks 12 and 24, only for patients in whom iron deficiency persisted
FERRIC-HF II	2019	Single centered	19	21	2 weeks	- Age ≥30 years - stable symptomatic chronic HF NYHA III and LVEF ≤45%, or if NYHA II then LVEF ≤40% within the preceding 6 months) - use of optimal HF drugs - Hb < 120 g/L in women and <130 g/L in men (anemic group) or ≥120 g/L in women and ≥130 g/L in men (nonanemic group)	skeletal muscle energetics	Ferritin<100 μg/L or 100–300 μg/L with transferrin saturation <20%	- Iron isomaltoside using the Ganzoni formula: body weight (kg) X 2.4 X (15 – patients Hb [g/dL]) + 500 mg (for stores) given as a single dose.
PRACTICE-ASIA-HF	2018	Multi-centered	24	25	12 weeks	- Age ≥21 years - clinical diagnosis of acute decompensated HF, regardless of LVEF - Iron deficiency	the difference in 6 MW test	Serum ferritin <300 ng/mL if TSAT <20%	- single dose of 1000 mg of IV FCM
EFFECT-HF	2017	Multi-centered	86	86	12 months	- Age ≥18 years - clinically stable mild to moderate chronic HF (NYHA II‒III), on optimal background therapy for HF - LVEF ≤45% - Plasma BNP >100 pg/mL or NT-proBNP >400 pg/mL - decreased exercise capacity; peak VO2 of 10 to 20 mL/kg/min. - ID	the change in peak VO2	Serum ferritin <100 ng/mL or a serum ferritin of 100 to 300 ng/mL in combination with TSAT <20%.	- IV iron Infusion of FCM that is equivalent to 500 or 1000 mg of iron) at day 0 and week 6. - At week 12, FCM was only administered (at a dose of 500 mg FCM) if serum ferritin was <100 ng/mL or if ferritin was 100 to 300 ng/mL with TSAT <20%
CONFIRM-HF	2015	Multi-centered	150	151	12 months	- Patients with stable ambulatory HF patients in NYHA II or III with LVEF ≤45% - BNP >100 pg/mL and/or NT-proBNP >400 pg/mL) - ID - Hb < 15 g/dl.	Change in 6 MW test	Serum ferritin level <100 ng/mL, or between 100 and 300 ng/mL if TSAT<20%	- FCM doses were between 500 and 2000 mg iron in the therapy phase (dosed at baseline and Week 6) - Maintenance FCM dosing of 500 mg iron at each of Weeks 12, 24, and 36, if ID was still present
IRON-HF	2013	Multi-centered	10	6	3 months	- Age ≥18 years - outpatients followed at a HF clinic - NYHA functional class II-IV, patients who are able to perform ergospirometry - LVEF<40% within the last 6 months - adequate baseline therapy for HF - Hemoglobin ≤12 g/dl and ≥9 g/dl - ID	peak VO2 assessed by ergospirometry.	- TSAT <20% and ferritin <500 μg/L	- IV iron sucrose 200 mg once a week for 5 weeks
FAIR-HF	2009	Multi-centered	304	155	24 weeks	- Ambulatory patients who had chronic HF with NYHA class II or III - LVEF ≤40% (for patients in NYHA class II) or ≤45% (for patients in NYHA class III) - Hb between 95 and 135 g per liter, and iron deficiency.	the self-reported Patient Global Assessment and NYHA functional class	- Ferritin level <100 μg per liter or 100–299 μg per liter, if the transferrin saturation was <20%	- Ferric Carboxymaltose equivalent to 200 mg of iron) given weekly until iron repletion was achieved (the correction phase) and then every 4 weeks during the maintenance phase, which started at week 8 or week 12
FERRIC-HF	2008	Multi-centered	24	11	18 weeks	- Age ≥21 years - symptomatic CHF: NYHA class II or III - exercise limitation as evidenced by a reproducible pVO2/kg ≤ 18 mL/kg/min - Hb < 12.5 g/dl (anemic group) or 12.5–14.5 g/dl (nonanemic group) - ID - LVEF≤ 45% - medically optimized CHF therapy for at least 4 weeks - normal folate and vitamin B12 levels	change in absolute pVO2 (ml/min)	- Ferritin< 100 g/L or 100–300 g/L with TSAT< 20%	- IV infusion of Iron sucrose (100 mg of iron) that was given weekly (therapeutic phase) unless ferritin was ≥500 ng/mL and then at weeks 4, 8, 12 and 16 (maintenance phase).
Toblli et al.	2007	Single centered	20	20	6 months	- LVEF ≤35% - NYHA functional class II to IV - Anemia Hb < 12.5 g/dl for men and <11.5 g/dl for women - ID - creatinine clearance ≤90 mL/min	- the effectiveness for improving hematologic and renal parameters - the change in the NT-proBNP level and inflammatory status by CRP	- Ferritin <100 ng/mL and/or TSAT ≤20%	IV Iron sucrose 200 mg weekly for 5 weeks.

HF: heart failure, NYHA: New York Heart Association; TSAT: transferrin saturation, HFrEF: heart failure with reduced ejection fraction, CRT: Cardiac resynchronization therapy, LVEF: left ventricular ejection fraction, MACE: Major adverse cardiovascular events, ACS: acute coronary syndrome, 6 MW test: 6 min walking test, BNP: brain natriuretic peptide, NT-proBNP: N-terminal pro hormone brain natriuretic peptide, ID: iron deficiency, Hb: hemoglobin, peak VO2: peak oxygen consumption, FCM: Ferric Carboxymaltose, CRP: C-reactive protein.

**Table 2:** Baseline characteristics of the studies population.

Studies	Groups	Age in years, mean ( ± SD)	Male %	BMI (kg/m2) [mean± SD]	Hb (g/dl) [mean± SD]	Ferritin (ng/ml) [mean± SD]	TSAT (%)	EF (%) [mean± SD]	NYHA class II (%)	NYHA class III (%)	6 MW test (m) [mean± SD]
Toblli et al., 2007	IV iron group	76 ± 7	–	28.7 ± 3.3	10.3 ± 0.6	73.0 ± 29.9	20 ± 1	31.3 ± 3.7	–	–	192.3 ± 60.9
	Control group	74 ± 8	–	29.0 ± 3.4	10.2 ± 0.5	70.6 ± 21.4	20 ± 1	30.8 ± 1.7	–	–	190.7 ± 56.1
FERRIC-HF 2008	IV iron group	64 ± 14	71	26 ± 5	12.6 ± 1.2	62 ± 37	20 ± 8	30 ± 7	54	46	–
	Control group	62 ± 11	73	28 ± 5	12.2 ± 1	88 ± 62	21 ± 9	29 ± 6	55	45	–
FAIR-HF 2009	IV iron group	67.8 ± 10.3	47.7	28.0 ± 4.8	11.9 ± 1.3	52.5 ± 54.5	17.7 ± 12.6	31.9 ± 5.5	17.4	82.6	274 ± 105
	Control group	67.4 ± 11.1	45.2	28.1 ± 5.1	11.9 ± 1.4	60.1 ± 66.5	16.7 ± 8.4	33.0 ± 6.1	18.7	81.3	269 ± 109
IRON-HF 2013	IV iron group	66.9 ± 8.3	66.7	–	11.2 ± 0.6	185 ± 146	18.9 ± 9.7	25.2 ± 8.6	–	–	–
	Control group	68.9 ± 10.1	66.7	–	10.9 ± 0.7	95 ± 128	13.5 ± 5.8	30.7 ± 7.4	–	–	–
CONFIRM-HF 2015	IV iron group	68.8 ± 9.5	55	28.3 ± 4.6	12.37 ± 1.41	57.0 ± 48.4	20.2 ± 17.6	37.1 ± 7.5	53	47	288 ± 98
	Control group	69.5 ± 9.3	51	29.1 ± 5.7	12.42 ± 1.30	57.1 ± 41.6	18.2 ± 8.1	36.5 ± 7.3	60	40	302 ± 97
EFFECT-HF 2017	IV iron group	63 ± 12	70	27.5 ± 5.0	12.9 ± 1.3	48	17.3	33 ± 9	71	29	–
	Control group	64 ± 11	80	26.9 ± 4.4	13.0 ± 1.5	53	18.1	31 ± 8	63	37	–
PRACTICE-ASIA-HF 2018	IV iron group	61.1 ± 10.8	75	–	11.6 ± 1.9	91.4 ± 80.4	15.7 ± 10.1	38.8 ± 17.5	–	–	252.4 ± 122.7
	Control group	64 ± 10	80	–	13.1 ± 1.3	84.1 ± 63.7	13.9 ± 6.8	33.2 ± 14.8	–	–	242.6 ± 66.8
FERRIC-HF II 2019	IV iron group	70 ± 12	76	29 ± 4	13.0 ± 1.5	34 (18–50)^a^	21 ± 8	37 ± 8	–	43	324 ± 79
	Control group	62 ± 13	68	30 ± 7	12.8 ± 2.0	59 (39–79)^a^	18 ± 10	37 ± 8	–	53	313 ± 67
AFFIRM-AHF 2020	IV iron group	71.2 ± 10.8	56	28.1 ± 5.6	12.3 ± 1.6	83.9 ± 62.2	15.2 ± 8.3	32.6 ± 9.6	46	49	–
	Control group	70.9 ± 11.1	55	28.0 ± 5.7	12.1 ± 1.6	88.5 ± 68.6	14.2 ± 7.5	32.7 ± 10.0	44	50	–
IDAN-HF 2021	IV iron group	65.5 ± 19.1	6.6	–	9.4 ± 2.6	–	–	44.6 ± 10.1	–	–	156.9 ± 72.5
	Control group	61.2 ± 16.1	23.3	–	11.6 ± 1.5	–	–	41.6 ± 8.2	–	–	254.7 ± 106.7
IRON-CRT 2021	IV iron group	72 ± 12	70	27 ± 5	13.3 ± 1.2	82 [38–106]^a^	18.8 ± 6.0	33 ± 8	59	41	–
	Control group	73 ± 9	66	27 ± 5	13.1 ± 1.3	81 [43–99]^a^	19.4 ± 7.0	34 ± 7	50	50	–
IRONMAN 2022	IV iron group	73.2 ± 6.9	75	28.5 ± 4.1	12·1 (11.2–12.8)^a^	49 (30.0–86.0)^a^	15 (11–20)^a^	32% (25–37)^a^	58	40	–
	Control group	73.5 ± 5.6	72	28.3 ± 3.6	12.1 (11.2–12.9)^a^	50 (30.0–85.0)^a^	15 (10–19)^a^	35% (26–38)^a^	56	42	–

IQR: interquartile range, SD: standard deviation, BMI: Body mass index, Hb: hemoglobin, EF: ejection fraction, TSAT: transferrin saturation, NYHA: New York Heart association, 6 MW: 6-min walk test, m: meter, IV: intravenous.

a Median (interquartile range).

**Table 3:** Baseline characteristics of the studies population.

Studies	Groups	NT-proBNP (pg/ml) [mean± SD]	CAD %	HTN %	DM %	HLD %	AF %	CVA %	Diuretics %	ACE inhibitors/ARBs/ARNI %	BB %	Digoxin %	MRA %
Toblli et al., 2007	IV iron group	255.9 ± 124.6	–	–	–	–	–	–	95	95	100	65	–
	Control group	267.5 ± 114.9	–	–	–	–	–	–	95	100	100	60	–
FERRIC-HF 2008	IV iron group	–	79	50	33	29	–	–	71	96	83	25	46
	Control group	–	73	45	36	45	–	–	73	91	100	18	55
FAIR-HF 2009	IV iron group	–	55.3 (MI)	79.7	30.6	47.4	30.9	7.9	92.1	92.4	86.2	15.1	–
	Control group	–	58.1 (MI)	82.6	23.9	45.2	28.4	5.8	90.3	91	83.2	16.1	–
IRON-HF 2013	IV iron group	–	22.2	22.2	33	–	22.2	–	–	–	–	–	–
	Control group	–	66.7	16.7	33.3	–	50	–	–	–	–	–	–
CONFIRM-HF 2015	IV iron group	2511 ± 5006	60 (MI)	87	25	65	44	14	88	100	89	19	–
	Control group	2600 ± 4555	60 (MI)	86	30	65	48	16	92	100	92	27	–
EFFECT-HF 2017	IV iron group	1576	67 (MI)	72	30	–	41	–	93	94	98	–	67
	Control group	1469	64 (MI)	65	37	–	48	–	95	90	98	–	72
PRACTICE-ASIA-HF 2018	IV iron group	–	50	87.5	62.5	83.3	–	–	87.5	79.1	100	–	29.2
	Control group	–	52	72	60	80	–	–	92	52	80	–	40
FERRIC-HF II 2019	IV iron group	1486 (245–2054)^a^	62	62	48	33	29	–	67	76	86	29	57
	Control group	462 (206– 855)^a^	58	64	53	37	21	–	63	89	84	21	63
AFFIRM-AHF 2020	IV iron group	4743 (2781–8128)^a^	41 (MI)	84	41	54	56	9	87	76	81	15	67
	Control group	4684 (2785–8695)^a^	39 (MI)	86	44	53	55	12	85	76	84	18	64
IDAN-HF 2021	IV iron group	–	–	93.3	–	–	–	–	–	–	–	–	–
	Control group	–	–	93.3	–	–	–	–	–	–	–	–	–
IRON-CRT 2021	IV iron group	2227 [299–2967]^a^	–	87	46	–	–	3	54	92	100	–	81
	Control group	1604 [767–2204]^a^	–	97	50	–	–	11	55	87	97	–	76
IRONMAN 2022	IV iron group	–	–	–	–	–	–	–	80	85	88	12	57
	Control group	–	–	–	–	–	–	–	82	88	90	11	54

NT-proBNP: N-terminal Plasma brain natriuretic peptide, CAD: coronary artery disease, MI: myocardial infarction, HTN: hypertension, DM: diabetes mellitus, HLD: hyperlipidemia, AF: atrial fibrillation, CVA: cerebrovascular accident, ACE: Angiotensin-converting enzyme, ARBs: angiotensin II receptor blockers, ARNI: angiotensin receptor/neprilysin inhibitor, BB: beta-blocker, MRA: Mineralocorticoid receptor antagonists.

a Median (interquartile range).

### 3.2 Main outcomes

**Fig. 2:**
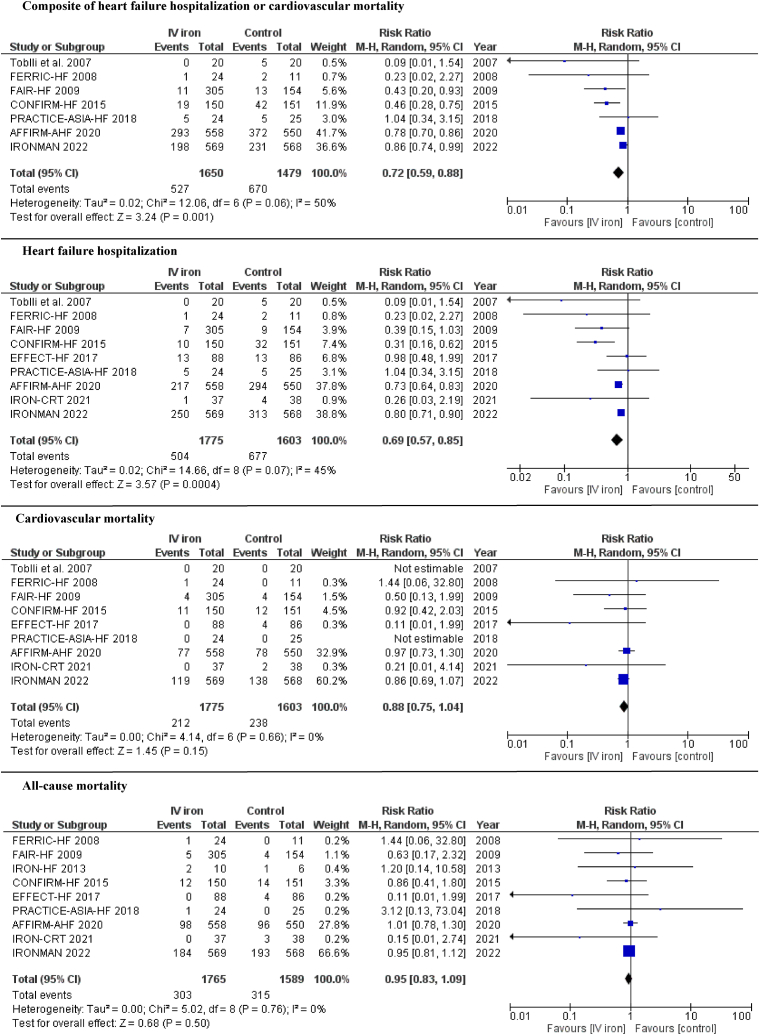
Forrest plot for composite of heart failure hospitalization or cardiovascular mortality, heart failure hospitalization, cardiovascular mortality, and all-cause mortality among the IV iron versus control groups.

IV iron administration was associated with a lower incidence of the composite of HF hospitalization or cardiovascular mortality compared with control (31.9% vs. 45.3%; RR 0.72; 95% confidence interval [CI] 0.59–0.88; I^2^ = 50%) (Fig. 2). This benefit was observed in acute (RR 0.83; 95% CI 0.73–0.93; I^2^ = 55%) and chronic HF (RR 0.52; 95% CI 0.31–0.88; I^2^ = 65%). The findings from the sensitivity analyses were consistent with the main analysis: studies using IV FCM (RR 0.63; 95% CI 0.42–0.93; I^2^ = 58%), excluding the study with the highest contribution to heterogeneity (RR 0.79; 95% CI 0.68–0.91; I^2^ = 31%), excluding studies with higher risk of bias (RR 0.54; 95% CI 0.33–0.89; I^2^ = 67%), including only studies with >100 sample size at each group (RR 0.73; 95% CI 0.60–0.89; I^2^ = 64%), and including only studies with follow-up >3 months (RR 0.72; 95% CI 0.58–0.88; I^2^ = 63%) (Supplemental Fig. 1 and Supplemental Fig. 2). IV iron administration was also associated with a reduction in individual outcome of HF hospitalization (28.4% vs. 42.2; RR 0.69; 95% CI 0.57–0.85; I^2^ = 45%) compared with control (Fig. 2). This benefit was observed in acute (RR 0.73; 95% CI 0.64–0.83; I^2^ = 0%) and chronic HF (RR 0.43; 95% CI 0.24–0.77; I^2^ = 35%). Sensitivity analyses of studies using IV FCM (RR 0.61; 95% CI 0.41–0.91; I^2^ = 47%), excluding the study with the highest contribution to heterogeneity (RR 0.76; 95% CI 0.67–0.85; I^2^ = 11%), excluding studies with higher risk of bias (RR 0.43; 95% CI 0.23–0.80; I^2^ = 62%), including only studies with >100 sample size at each group (RR 0.68; 95% CI 0.55–0.85; I^2^ = 69%), and including only studies with follow-up >3 months (RR 0.69; 95% CI 0.56–0.86; I^2^ = 59%) showed similar results (Supplemental Fig. 2 and Supplemental Fig. 3). Meta-regression analyses showed no effect modification of the composite of HF hospitalization or cardiovascular mortality based on age (p = 0.90), male sex (p = 0.36), hemoglobin level (p = 0.05), ferritin level (p = 0.25), or LVEF (p = 0.75). Similarly, no effect modification was observed for individual outcome of HF hospitalization based on age (p = 0.90), male sex (p = 0.05), hemoglobin level (p = 0.07), ferritin level (p = 0.83), or LVEF (p = 0.79). Inspection of the funnel plot suggested no evidence of publication bias (Supplemental Fig. 4).

### 3.3 Other outcomes

**Fig. 3:**
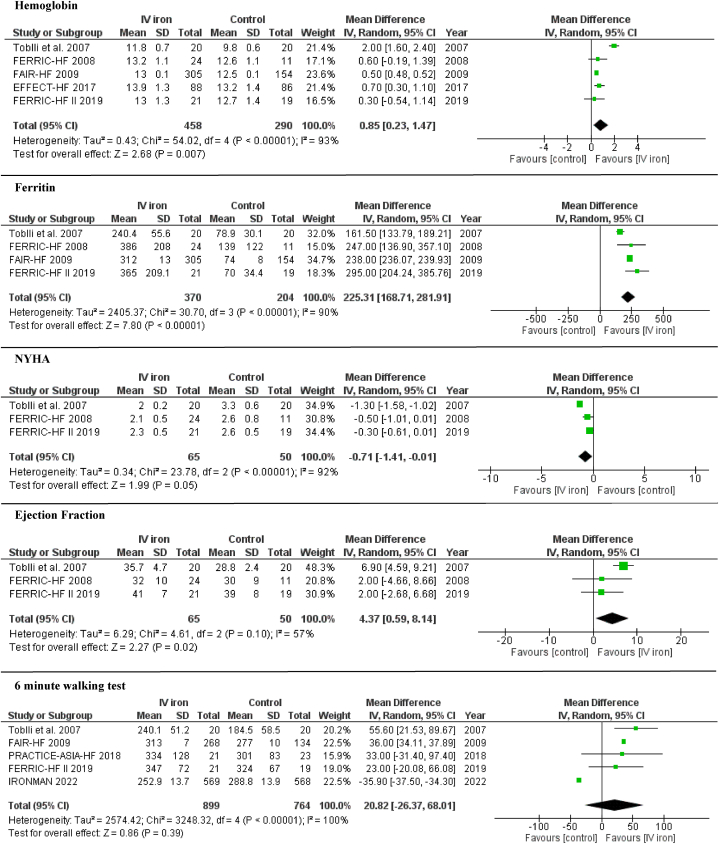
Forrest plot for hemoglobin level and ferritin level, NYHA score, ejection fraction and 6-min walking test among the IV iron versus control groups.

There was no difference between IV iron and control groups in cardiovascular mortality (RR 0.88; 95% CI 0.75–1.04; I^2^ = 0%) and all-cause mortality (RR 0.95; 95% CI 0.83–1.09; I^2^ = 0%). Compared with control, IV iron administration was associated with higher hemoglobin levels (MD 0.85; 95% CI 0.23–1.47; I^2^ = 93%), higher ferritin levels (MD 225.31; 95% CI 168.71–281.91; I^2^ = 90%), lower NYHA class (MD -0.71; 95% CI -1.41 to −0.01; I^2^ = 92%) and higher EF (MD 4.37; 95% CI 0.59–8.14; I^2^ = 57%). There was no difference in 6-min walk test (MD 20.82; 95% CI -26.37 – 68.01; I^2^ = 100%) (Fig. 2, Fig. 3).

## 4 Discussion

In this meta-analysis of 12 RCTs including 3,492 patients [3,7,[9], [10], [11], [12], [13], [14], [15], [16], [17], [18]], we evaluated the efficacy of IV iron administration in patients with HF and concomitant ID. The main findings of this study are: 1) patients with HF and ID who received IV iron had lower incidence of the composite of HF hospitalization or cardiovascular mortality driven by a lower incidence of HF hospitalizations; 2) this benefit was observed in both acute and chronic HF, 3) meta-regression suggested no effect modification based on age, sex, hemoglobin level, ferritin level or LVEF; 4) there was no difference in all-cause and cardiovascular mortality in patients with HF and ID who received IV iron; 5) IV iron administration in patients with HF and ID demonstrated improvement in hemoglobin and ferritin levels, higher EF and lower NYHA class compared with control.

Treatment of ID among patients with HF has been evaluated previously via high dose oral iron, however, studies showed no significant clinical benefits, and this was attributed to poor absorption of oral iron formulations in patients with HF [34]. The role of IV iron administration in patients with HF and ID has been explored in multiple randomized studies and prior meta-analyses [3,7,[9], [10], [11], [12], [13], [14], [15], [16], [17], [18], [19], [20], [21], [22], [23], [24], [25], [26], [27], [28], [29], [30]]. The results of these studies suggested beneficial impact for IV iron on improving the symptoms and functionality of patients with HF. However, these studies showed inconsistencies regarding the impact of IV iron on reducing hard clinical outcomes including HF hospitalizations, cardiovascular or all-cause mortality. Prior meta-analyses demonstrated a potential reduction in cardiovascular hospitalizations or HF hospitalizations with IV iron [[19], [20], [21], [22], [23], [24], [25], [26], [27],^29^]. In addition, Mei et al. compared both oral and IV iron in patients with ID and HF. It showed that both IV and oral iron reduced all-cause death and HF hospitalization, but only IV iron improved the exercise capacity and quality of life [28]. A recent meta-analysis by Salah et al. demonstrated that IV iron in patients with HFrEF reduced the composite risk of first hospitalization for HF or CV mortality in addition to a reduced risks of first hospitalization and recurrent HF hospitalizations, but showed no effect on CV morality or all-cause mortality [30]. The IRONMAN trial is the most recent and largest RCT that evaluated the role of IV iron among patients with HF. IRONMAN RCT study of 1,137 patients showed no statistically significant difference in the primary endpoint of HF hospitalization or all-cause mortality between the IV iron and control groups during a median follow-up of 2.7 years [18].

In the current meta-analysis, we included the totality of available randomized data, including the IRONMAN trial. Our analysis demonstrated the beneficial impact of IV iron in reducing the composite of HF hospitalization or cardiovascular mortality and individual outcome of HF hospitalization. While our analysis suggested a numerical reduction in cardiovascular mortality with IV iron, there was no statistically significant difference in cardiovascular or all-cause mortality among both groups. Importantly, we have conducted subgroup and several exploratory analyses which demonstrated consistent beneficial impact of IV iron irrespective of the clinical presentation (i.e., acute versus chronic HF), and without heterogeneity of treatment effect across age, sex, hemoglobin level, ferritin level or LVEF. These findings suggest that the clinical benefits with IV iron could be still attained through initiation of IV iron during inpatient or outpatient settings, and across a broad spectrum of patients with HF at various severities of ID. Our study results are discordant with those of the IRONMAN trial. This might be related to the limitations and restrictions in the IRONMAN trial that was conducted during the COVID-19 pandemic, which affected obtaining blood tests to assess for iron deficiency and the need for redosing with IV iron therapy [18].

Despite the advances in current guideline-directed medical therapies for HF, recurrent HF hospitalizations remains to be a major burden on the healthcare system, that is associated with high morbidity and mortality. Our study results confirm the viable role for IV iron in the current armamentarium for treating patients with HF. However, our updated analysis still failed to demonstrate a significant survival benefit with IV iron among patients with HF, despite including the largest and most recent RCTs. It is plausible that our study is still underpowered to detect survival benefits, and larger RCTs are still warranted to interrogate the possible survival benefit with IV iron.

Several mechanisms are proposed to be the etiology of ID in patients with HF. Hepcidin plays an important role in the regulation of ferroportin which has a major role in iron uptake. Heart failure, a known state of inflammation, results in an increase of hepcidin. This in turn results in down regulation of ferroportin and as a result causes less iron absorption [35]. Furthermore, gut wall edema and resultant decreased absorption play an important role [36]. By contrast, ID has been proposed to be a risk factor for worsening HF [37]. Iron is a metal cofactor in the production of mitochondrial enzymes which is essential in energy production and cellular metabolism in the myocardium [[37], [38], [39]]. Moreover, iron has a role in oxygen delivery, storage (in the form of myoglobin) and oxidative metabolism in the cardiac muscle [40]. As a result, ID can cause abnormal cellular metabolism and mitochondrial dysfunction which may predispose to worsening HF [41,42]. IV iron administration can improve oxygen metabolism and transport, in addition to increase oxygen carrying capacity through increase in hemoglobin especially in cells with high oxygen demands [26]. Previous studies demonstrated that IV iron in patients with HFrEF could replenish the intra-myocardial iron content which could help improve left ventricular function [43,44]. It was previously recommended that all patients with HF undergo screening for iron deficiency with baseline labs including complete blood count, ferritin, and transferrin saturation [45].

This meta-analysis encompasses the totality of available randomized trials regarding the efficacy of IV iron administration in patients with HF and ID. Our results demonstrated that the use of IV iron in patients with HF and ID regardless of anemia decreases the composite of HF hospitalization or cardiovascular mortality, and the individual risk of recurrent HF hospitalization. Nevertheless, this study had several limitations. First, there were variabilities among the included studies in the IV iron formulas, which may impact the treatment effect. There were also variabilities in the LVEF of included patients. To mitigate these variabilities, we have conducted exploratory analyses by including studies using consistent IV iron formula (i.e, FCM), and found similar results. Also, a meta-regression analysis showed no effect modification of the primary outcome based on LVEF. Second, despite including the totality of randomized data, our analysis might have been underpowered for some of the secondary outcomes, such as all-cause mortality and cardiovascular mortality. Third, there were moderate degree of heterogeneity in the main study outcomes, so we conducted stepwise sensitivity analyses to evaluate the source of heterogeneity by excluding one study at a time, then we excluded the study with the highest contribution to heterogeneity. Fourth, most of the included studies had very low sample size and limited follow-up. We have conducted sensitivity analyses including only studies with >100 sample size at each group, and including studies with follow-up >3 months, both showed similar results on the primary outcome. Fifth, our study included non-blinded and single center studies, so we have conducted a sensitivity analysis excluding studies with higher risk of bias that have showed similar results. Finally, there was lack of patient level data, so we could not ascertain which group drives the most benefit from IV iron therapy.

## 5 Conclusions

In this meta-analysis of RCTs, IV iron administration among patients with HF and ID was associated with a lower incidence of the composite of HF hospitalization or cardiovascular mortality, an effect that was driven by a reduction in the incidence of HF hospitalizations. The beneficial outcomes with IV iron were observed across a broad spectrum of patients with HF, regardless of the clinical setting (i.e., inpatient versus outpatient), age, sex, LVEF, hemoglobin level or ferritin level. There was no significant difference in the incidence of all-cause mortality or cardiovascular mortality among both study groups. Further efforts may be considered towards widespread testing and treatment of ID in patients with HF.

## Production notes

### Author contribution statement

Mohamed Hamed, Sheref A. Elseidy, Mamas A. Mamas, Islam Y. Elgendy & Ayman Elbadawi: Conceived and designed the experiments; Performed the experiments; Analyzed and interpreted the data; Contributed analysis tools or data; Wrote the paper.

Ravi Thakker, Hend Mansoor, & Houman Khalili: Performed the experiments; Contributed analysis tools or data; Wrote the paper.

Asmaa Ahmed & Amr Mohsen: Performed the experiments; Analyzed and interpreted the data; Wrote the paper.

Subhash Banerjee & Dharam J. Kumbhani: Analyzed and interpreted the data; Wrote the paper.

### Data availability statement

Data will be made available on request.

## Declaration of competing interest

The authors declare that they have no known competing financial interests or personal relationships that could have appeared to influence the work reported in this paper.
