# Supplementary material for: Intravenous iron therapy among patients with heart failure and iron deficiency: An updated meta-analysis of randomized controlled trials
Source: Heliyon. 2023 Jun 15;9(6):e17245. doi: 10.1016/j.heliyon.2023.e17245 (PMC10293724; doi:10.1016/j.heliyon.2023.e17245)
Supplement: Multimedia component 1 [file mmc1.pptx]

## Slide 1
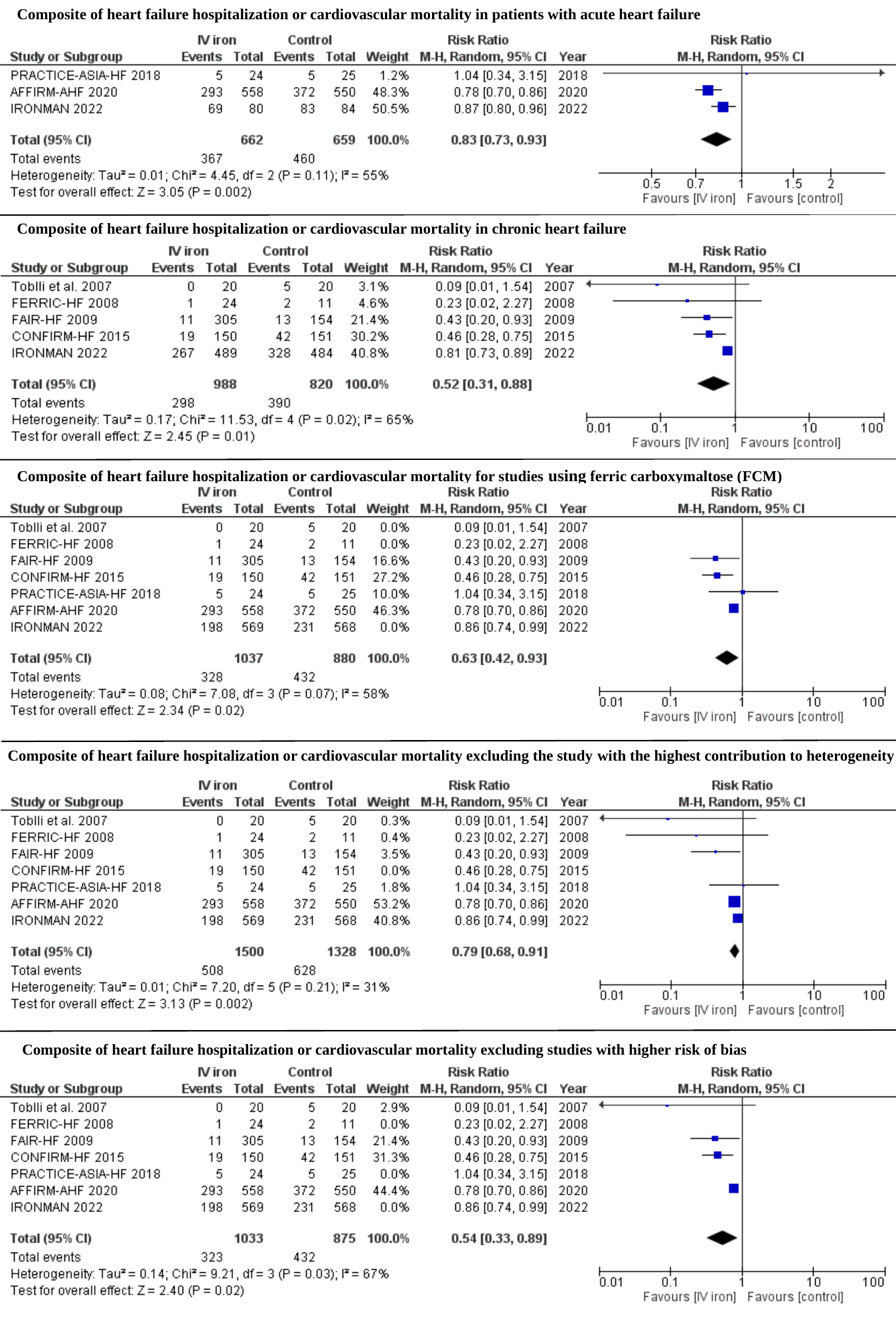

Composite of heart failure hospitalization or cardiovascular mortality in patients with acute heart failure
Composite of heart failure hospitalization or cardiovascular mortality in chronic heart failure
Composite of heart failure hospitalization or cardiovascular mortality for studies using ferric carboxymaltose (FCM)
Composite of heart failure hospitalization or cardiovascular mortality excluding the study with the highest contribution to heterogeneity
Composite of heart failure hospitalization or cardiovascular mortality excluding studies with higher risk of bias
