# Supplementary material for: Intravenous iron therapy among patients with heart failure and iron deficiency: An updated meta-analysis of randomized controlled trials
Source: Heliyon. 2023 Jun 15;9(6):e17245. doi: 10.1016/j.heliyon.2023.e17245 (PMC10293724; doi:10.1016/j.heliyon.2023.e17245)
Supplement: Multimedia component 2 [file mmc2.pptx]

## Slide 1
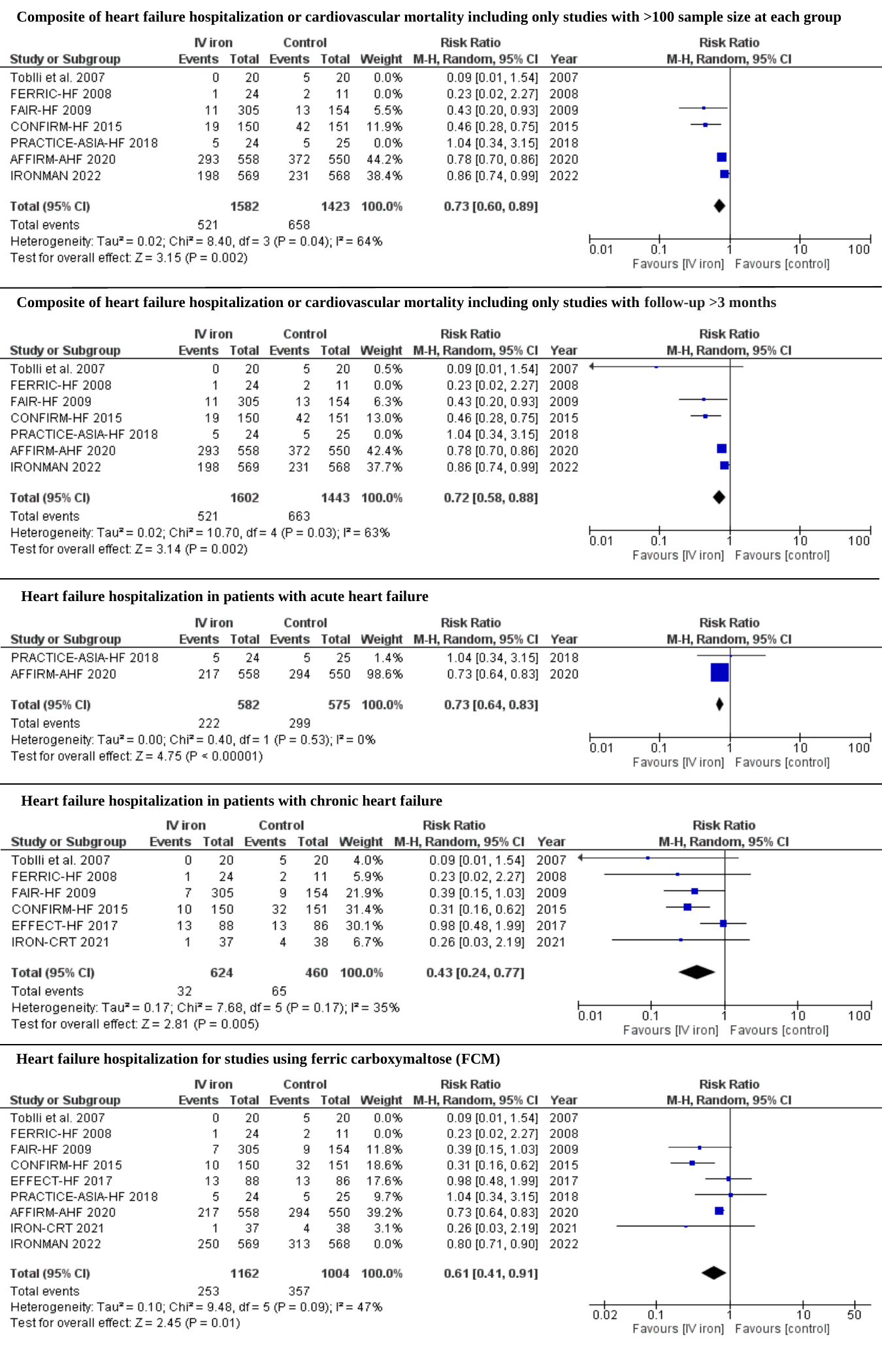

Composite of heart failure hospitalization or cardiovascular mortality including only studies with >100 sample size at each group
Composite of heart failure hospitalization or cardiovascular mortality including only studies with follow-up >3 months
Heart failure hospitalization in patients with acute heart failure
Heart failure hospitalization in patients with chronic heart failure
Heart failure hospitalization for studies using ferric carboxymaltose (FCM)
