# Supplementary material for: Intravenous iron therapy among patients with heart failure and iron deficiency: An updated meta-analysis of randomized controlled trials
Source: Heliyon. 2023 Jun 15;9(6):e17245. doi: 10.1016/j.heliyon.2023.e17245 (PMC10293724; doi:10.1016/j.heliyon.2023.e17245)
Supplement: Multimedia component 3 [file mmc3.pptx]

## Slide 1
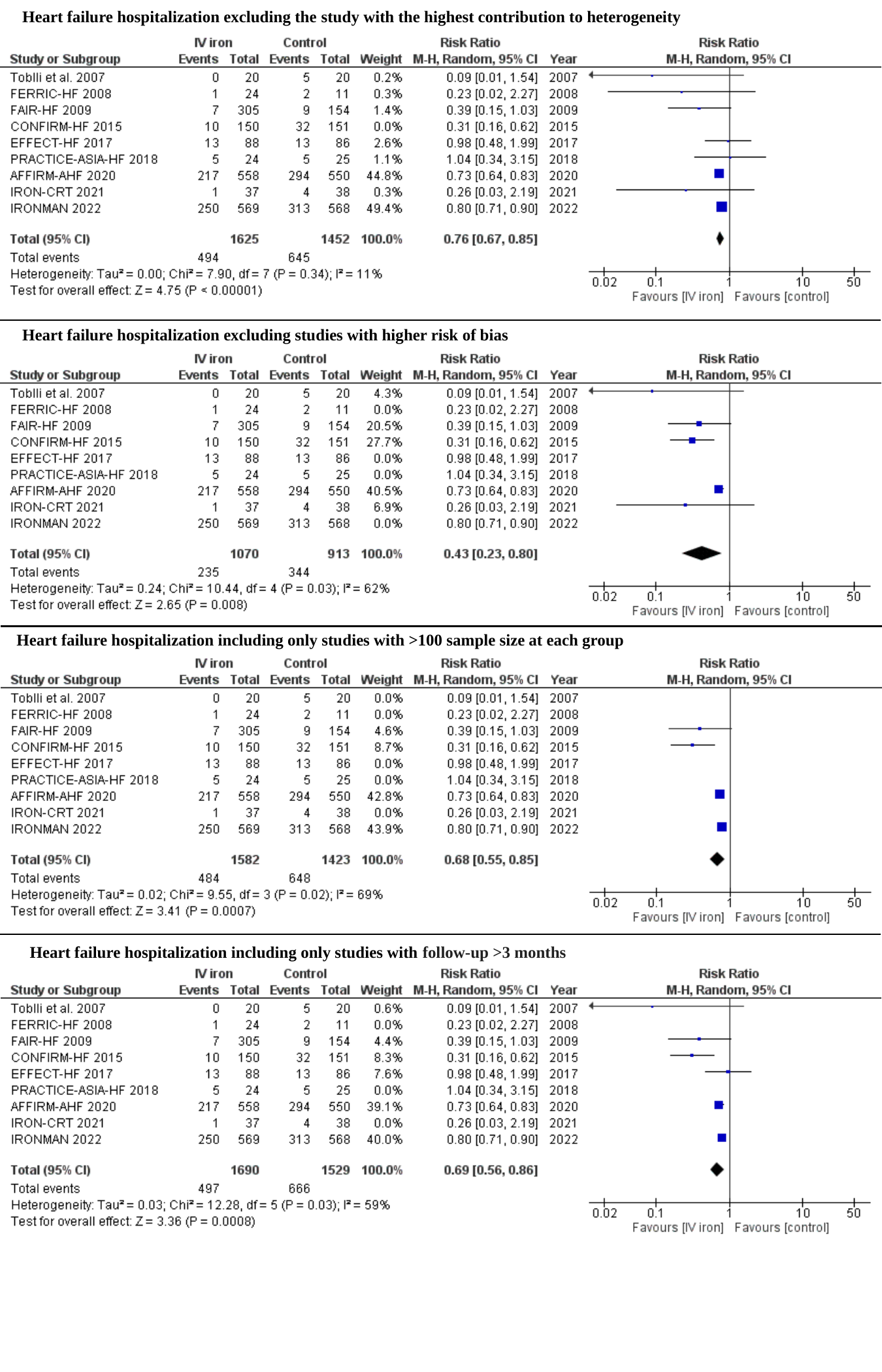

Heart failure hospitalization excluding the study with the highest contribution to heterogeneity
Heart failure hospitalization excluding studies with higher risk of bias
Heart failure hospitalization including only studies with >100 sample size at each group
Heart failure hospitalization including only studies with follow-up >3 months
