# Supplementary material for: Intravenous iron therapy among patients with heart failure and iron deficiency: An updated meta-analysis of randomized controlled trials
Source: Heliyon. 2023 Jun 15;9(6):e17245. doi: 10.1016/j.heliyon.2023.e17245 (PMC10293724; doi:10.1016/j.heliyon.2023.e17245)
Supplement: Multimedia component 4 [file mmc4.pptx]

## Slide 1
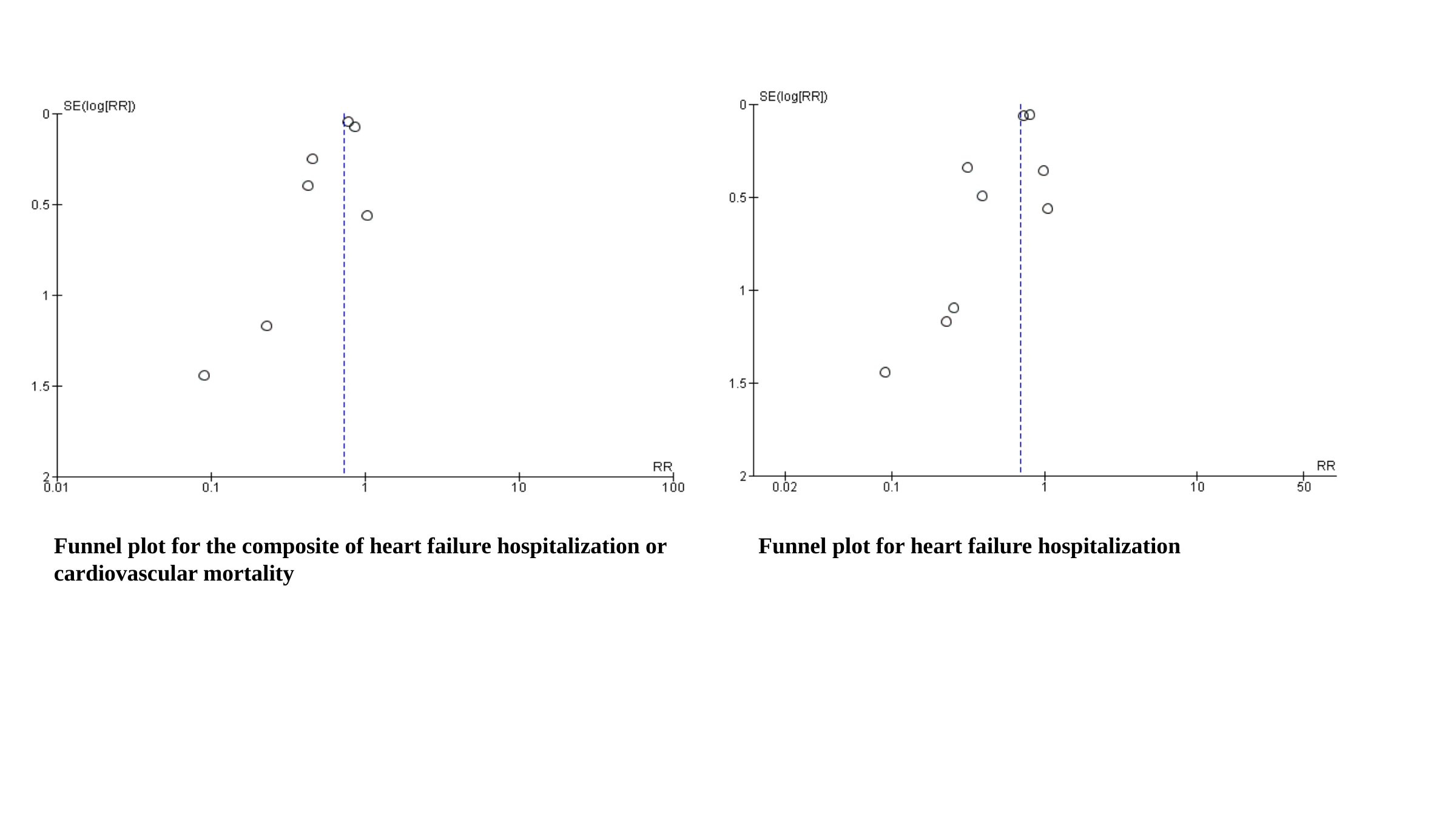

Funnel plot for the composite of heart failure hospitalization or cardiovascular mortality
Funnel plot for heart failure hospitalization
