# Supplementary material for: Intravenous iron therapy among patients with heart failure and iron deficiency: An updated meta-analysis of randomized controlled trials
Source: Heliyon. 2023 Jun 15;9(6):e17245. doi: 10.1016/j.heliyon.2023.e17245 (PMC10293724; doi:10.1016/j.heliyon.2023.e17245)
Supplement: Multimedia component 5 [file mmc5.docx]

| **Section and Topic** | **Item #** | **Checklist item** | **Pages where item**  **is reported** |
| --- | --- | --- | --- |
| **TITLE** | | |  |
| Title | 1 | Identify the report as a systematic review. | 1 |
| **ABSTRACT** | | |  |
| Abstract | 2 | See the PRISMA 2020 for Abstracts checklist. | 2 |
| **INTRODUCTION** | | |  |
| Overview | 3 | Overview on the recent data and current evidence | 3 |
| Rationale | 4 | Describe the rationale for the review in the context of existing knowledge. | 3 |
| Objectives | 5 | Provide a clear statement of the objectives of the review | 3 |
| **METHODS** | | |  |
| Data sources and Search strategy | 6 | Provide the full search strategies for all databases, websites, scientific sessions including search terms used in the systemic review. | 3-4 |
| Eligibility criteria | 7 | Specify the inclusion and exclusion criteria for the review and how studies were grouped for the syntheses. | 4 |
| Data collection process | 8 | Specify the number of investigators and data extracted, whether they work independently, and the process of confirming data from study investigators | 4 |
| Outcomes | 9 | Identify the study outcomes including definition of endpoints | 4 |
| Study risk of bias assessment | 10 | Provides how risk of bias was evaluated in the included studies and the criteria used in the evaluation | 4 |
| Statistical analysis | 11 | Describe methods used to synthesize results, presence, and degree of heterogeneity. It also identifies the software package used in the analysis | 5 |
| Certainty assessment | 12 | Describe methods used to assess certainty for outcomes | 5 |
| **RESULTS** | | |  |
| Study selection | 13 | Describe the results of the search and selection process from the number identified to the number of studies included in the review, then it is included in a flow diagram | Page 5, Figure 1 |
| Study characteristics | 14 | Cite each included study and present its characteristics | 5-6 |
| Risk of bias in included studies | 15 | Provides assessments of risk of bias for each included study | 5-6 |
| outcomes | 16 | Provides the study outcomes using appropriate structured tables or plots. | 6-7 |

| **Section and Topic** | **Item #** | **Checklist item** | **Location where item**  **is reported** |
| --- | --- | --- | --- |
| **DISCUSSION** | | |  |
| Discussion | 17a | Provide a general interpretation of the results | 7 |
|  | 17b | Compare the review to previously published reviews and their limitations. It also provides an explanation on the importance of this review in overcoming those limitations. | 7-8 |
|  | 17c | Describe possible reasons of the review outcomes by providing previous evidence. | 8-9 |
|  | 17d | Discuss implications of the review results in current practice and future research | 9 |
|  | 17e | Discuss possible limitations of the current review | 9-10 |
| **Conclusion** | | |  |
| Conclusion | 18 | Summarize review outcome | 10 |
| **OTHER INFORMATION** | | |  |
| Registration and protocol | 19 | Provided registration information of the review and how to assess review protocol | 4 |

*From:* Page MJ, McKenzie JE, Bossuyt PM, Boutron I, Hoffmann TC, Mulrow CD, et al. The PRISMA 2020 statement: an updated guideline for reporting systematic reviews. BMJ 2021;372:n71. doi: 10.1136/bmj.n71

For more information, visit: <http://www.prisma-statement.org/>
