# Supplementary material for: Intravenous iron therapy among patients with heart failure and iron deficiency: An updated meta-analysis of randomized controlled trials
Source: Heliyon. 2023 Jun 15;9(6):e17245. doi: 10.1016/j.heliyon.2023.e17245 (PMC10293724; doi:10.1016/j.heliyon.2023.e17245)
Supplement: Multimedia component 6 [file mmc6.docx]

Supplemental Table 2: Risk of bias of the individual studies by Cochrane risk assessment tool

|  | Toblli et al. 2007 | FERRIC-HF 2008 | FAIR-HF 2009 | IRON-HF 2013 | CONFIRM-HF 2015 | EFFECT-HF 2017 | PRACTICE-ASIA-HF 2018 | FERRIC-HF II 2019 | AFFIRM-AHF 2020 | IDAN‑HF 2021 | IRON-CRT 2021 | IRONMAN 2022 |
| --- | --- | --- | --- | --- | --- | --- | --- | --- | --- | --- | --- | --- |
| **Random sequence generation** *(Selection bias)* | 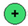 | 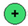 | 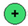 | 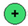 | 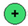 | 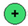 | 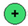 | 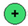 | 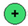 | 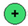 | 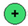 | 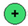 |
| **Allocation concealment** *(Selection bias)* | 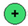 | 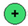 | 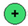 | 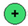 | 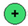 | 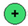 | 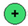 | 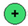 | 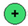 | 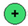 | 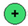 | 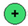 |
| **Blinding of participants and personnel** *(Performance bias)* | 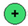 | 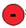 | 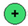 | 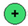 | 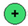 | 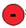 | 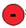 | 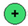 | 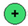 | 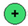 | 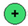 | 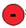 |
| **Blinding of outcome assessment** *(Detection bias)* | 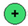 | 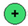 | 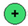 | 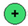 | 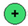 | 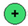 | 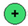 | 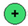 | 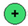 | 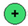 | 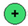 | 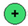 |
| **Incomplete outcome data** *(Attrition bias)* | 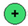 | 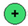 | 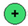 | 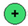 | 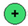 | 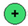 | 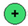 | 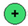 | 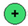 | 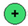 | 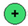 | 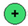 |
| **Selective reporting** *(Reporting bias)* | 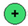 | 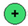 | 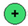 | 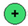 | 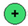 | 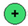 | 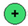 | 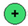 | 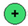 | 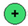 | 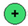 | 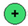 |
| **Other sources of bias** | 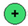 | 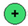 | 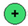 | 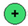 | 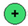 | 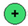 | 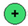 | 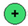 | 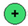 | 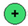 | 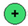 | 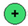 |


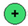
 = Low risk of bias
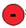
 = Risk of bias
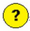
 = Unclear
